# The value of pretreatment serum butyrylcholinesterase level as a novel prognostic biomarker in patients with cervical cancer treated with primary (chemo-)radiation therapy

**DOI:** 10.1007/s00066-019-01430-z

**Published:** 2019-02-08

**Authors:** Nina Poetsch, Alina Sturdza, Stefanie Aust, Stephan Polterauer, Christoph Grimm, Richard Schwameis, Richard Pötter, Heinz Koelbl, Alexander Reinthaller, Veronika Seebacher

**Affiliations:** 1 Department for Gynecology and Gynecologic Oncology, Gynecologic Cancer Unit, Comprehensive Cancer Centre, Medical University of Vienna, Waehringer Guertel 18–20, 1090 Vienna, Austria; 2 Department for Radiotherapy, Gynecologic Cancer Unit, Comprehensive Cancer Centre, Medical University of Vienna, Waehringer Guertel 18-20, Vienna, 1090 Austria

**Keywords:** Cholinesterase, Cervix cancer, Prognosis, Treatment response, Survival, Cholinesterase, Gebährmutterhalskrebs, Prognose, Therapieansprechen, Überleben

## Abstract

**Background:**

Deficiency in butyrylcholinesterase (BChE), a condition commonly noticed in liver damage, inflammation, and malnutrition, has previously been associated with impaired prognosis in different malignancies. The aim of the present study was to investigate the value of pretreatment serum BChE levels as a prognostic biomarker in patients with cervical cancer treated with primary (chemotherapy-[chemo-])radiation therapy.

**Methods:**

We retrospectively evaluated data of a consecutive series of patients with cervical cancer treated with primary (chemo-)radiation therapy between 1998 and 2015. Pretreatment serum BChE levels were correlated with clinico-pathological parameters and response to treatment. Uni- and multivariate survival analyses were performed to assess the association between decreased serum BChE levels and progression-free (PFS), cancer-specific (CSS), and overall survival (OS).

**Results:**

A total of 356 patients were eligible for inclusion into the present study. The median (IQR) pretreatment serum BChE level was 6180 (4990–7710) IU/l. Lower serum BChE levels were associated with lower BMI (*p* < 0.001), advanced tumor stage (*p* = 0.04), poor treatment response (*p* = 0.002), the occurrence of disease recurrence (*p* = 0.003), and the risk of death (*p* < 0.001). In uni- and multivariate analyses, low pretreatment serum BChE levels were independently associated with shorter PFS (HR 1.8 [1.2–2.6]; *p* = 0.002), CSS (HR 2.2 [1.4–3.5], *p* < 0.001), and OS (HR 2.0 [1.4–2.9]; *p* < 0.001).

**Conclusions:**

Low pretreatment serum BChE levels are associated with advanced tumor stage and poor response to treatment, and serve as an independent prognostic biomarker for shorter PFS, CSS, and OS in patients with cervical cancer treated with primary (chemo-)radiation therapy.

## Zusammenfassung

**Hintergrund:**

Für erniedrigte Butyrylcholinesterase(BChE)-Spiegel, die üblicherweise im Rahmen von Leberschäden, Entzündungsreaktionen oder Mangelernährung auftreten, wurden kürzlich auch Assoziationen mit schlechterem Überleben in verschiedenen onkologischen Erkrankungen beschrieben. Das Ziel dieser Studie war, die prognostische Bedeutung von prätherapeutischen BChE-Serumspiegeln für das Überleben von Zervixkarzinompatientinnen unter primärer (Chemotherapie- [Chemo-])Radiotherapie zu untersuchen.

**Methoden:**

Prätherapeutische BChE-Werte im Serum aller Zervixkarzinompatientinnen, die im Zeitraum von 1998 bis 2015 mit einer primären (Chemo‑)Radiotherapie behandelt wurden, wurden retrospektiv erhoben und mit klinisch-pathologischen Parametern sowie dem Therapieansprechen korreliert. In uni- und multivariaten Überlebensanalysen wurde der Zusammenhang zwischen erniedrigten BChE-Spiegeln und progressionsfreiem (PFS), tumorspezifischem (CSS) und Gesamtüberleben (OS) untersucht.

**Ergebnisse:**

Insgesamt konnten 356 Patientinnen mit einem medianen prätherapeutischen BChE-Spiegel (IQR) von 6180 IU/l (Spanne 4990–7710 IU/l) in die Analyse einbezogen werden. Die medianen BChE-Serumspiegel waren in Patientinnen mit niedrigerem Body-Mass-Index (*p* < 0,001), fortgeschrittenem Tumorstadium (*p* = 0,04), schlechtem Therapieansprechen (*p* = 0,002) und dem Auftreten eines Rezidivs signifikant erniedrigt (*p* = 0,003). In uni- und multivariaten Überlebensanalysen waren niedrigere BChE-Spiegel (<6180 IU/l) mit kürzerem PFS (HR 1,8 [1,2–2,6]; *p* = 0,002), CSS (HR 2,2 [1,4–3,5], *p* < 0,001) und OS (HR 2,0 [1,4–2,9]; *p* < 0,001) assoziiert.

**Schlussfolgerung:**

Erniedrigte prätherapeutische BChE-Spiegel sind mit fortgeschrittenem Tumorstadium und schlechterem Therapieansprechen assoziiert und eignen sich als unabhängige Prognoseparameter für kürzeres PFS, CSS und OS bei Zervixkarzinompatientinnen unter primärer (Chemo-)Radiotherapie.

## Introduction

Despite substantial reductions in incidence rates through screening programs, cervical cancer still represents a major public health problem, with approximately 54,517 women being newly diagnosed and 24,874 dying every year in Europe [1]. While cervical cancer confined to the cervix can be treated with surgery alone, for patients with locally advanced cervical cancer (LACC) or lymph node metastases, primary chemoradiation therapy consisting of external beam radiation therapy (EBRT), utero-vaginal brachytherapy, and concurrent platinum-based chemotherapy is currently considered standard of care [2–4]. This treatment regime shows good long-term results for disease-free survival, with treatment-related toxicities ≥ grade 2 in 33%, mainly involving bladder and bowel [5]. The contribution of adding adjuvant chemotherapy to chemoradiation for patients with LACC is currently being addressed in a randomized phase III study (The OUTBACK trial, NCT01414608). Adjuvant hysterectomy following primary chemoradiation showed promising results in patients with residual disease [6]. Estimation of a patient’s oncological outcome is essential in order to adequately tailor treatment and to help in selecting patients as candidates for clinical trials. Numerous clinical and pathological factors have been evaluated for their value in predicting prognosis in cervical cancer. Based on identified factors, such as histologic subtype, ethnicity, performance status, tumor size, tumor stage, grade, and the presence of lymph node metastases, nomograms have been developed to estimate the individual patient’s survival rate as accurately as possible [7, 8]. Chronic inflammation is generally understood to play a critical role in tumor initiation and promotion [9]. Therefore, an inflammation-based index using serum c-reactive protein (CRP) and albumin values was shown to correlate with survival in patients with hepatocellular carcinoma undergoing stereotactic body radiotherapy [10]. Moreover, inflammatory reactions leading to metabolic alterations are strongly linked with muscle wasting, a key symptom of cancer cachexia [11]. Several serological biomarkers reflecting a state of inflammation and malnutrition have been related to the oncological outcome of patients with cervical cancer [12–16]. Serum albumin levels, CRP, neutrophils, and platelet counts, for instance, seem to be associated with tumor stage and survival in patients with cervical cancer [12, 15–17]. Butyrylcholinesterase (BChE), an alpha-glycoprotein synthesized and secreted into blood by the liver, is a non-specific cholinesterase enzyme found in most tissues, including the nervous system, small intestine, and adipose tissue. BChE hydrolyzes various exogenous choline esters, such as medication used in anesthesiology [18]. Elevated BChE activities are observed in obesity, diabetes, uremia, hyperthyroidism, and hyperlipidemia [19]. On the contrary, decreased serum BChE levels are found in acute and chronic liver damage, liver metastases, and cirrhosis as a biochemical marker of organ damage and impaired synthetic function. Moreover, decreased serum BChE levels have been observed in various clinical conditions, such as stress, chronic inflammation, and malnutrition [20]. In addition, low serum BChE levels seem to be indicative of advanced tumor stage and poor prognosis in various forms of cancer, including gastric [21], renal [22], upper urinary tract [23], prostate [24], and head and neck cancer [25, 26]. In cervical cancer, two studies in small series of patients have reported on an association between low serum BChE levels and advanced tumor stage, and have suggested a correlation between the increase in serum BChE levels and a response to radiotherapy [25, 27]. The aim of the present study was to investigate the prognostic value of pretreatment serum BChE levels for survival in patients with cervical cancer treated with primary (chemotherapy-[chemo-])radiation therapy.

## Patients and methods

### Patients

Data of all patients treated with primary (chemo-)radiation therapy for either locally advanced cervical cancer (FIGO stage IIB–IVB) or early stage cervical cancer (FIGO stage ≤ IIA) with lymph node metastases at our institution between 1998 and 2015 were evaluated. Patients with early stage cervical cancer who received primary surgical treatment, those treated with chemotherapy alone, and patients with additional, coexisting malignant disease were excluded from analysis. Patients’ records were reviewed to identify those with serological measurements of BChE available prior to treatment. These patients were eligible for analyses within the present study. The study was approved by the institutional review board (Project # 2160/2016). The patient data were de-identified and handled in accordance with ethical standards of good scientific practice and the Helsinki Declaration.

### Clinical management

Diagnosis of cervical cancer was established by cervical biopsy. Subsequently, patients were staged according to the 1995 International Federation of Gynecology and Obstetrics (FIGO) classification system [28], more recently according to the revised version of 2009 [29]. To harmonize data, the 2009 FIGO staging system was used for all patients in the present study. Clinical staging was complemented by imaging studies, such as magnetic resonance imaging (MRI), computed tomography (CT), or combined 2‑deoxy-2-[^18^F]-fluoro-D-glucose positron-emission tomography ([^18^F]FDG PET)-CT scans, to guide treatment. Depending on the year of treatment and the presence of comorbidities, lymph node status was either evaluated by imaging methods or by surgical staging of pelvic and/or periaortic lymph nodes. Locally advanced cervical cancer (including patients with involved periaortic lymph nodes) or early stage cervical cancer with lymph node metastases was treated by concurrent chemoradiation therapy including image-guided adaptive brachytherapy (IGABT) [30–32]. Of note, chemotherapy concurrent to radiation therapy was administered starting in 1999. Metastatic cervical cancer (FIGO IVB, beyond the presence of periaortic lymph node metastases) was treated by individually tailored radiation and chemotherapy, as recommended by the multidisciplinary tumor board. Response to treatment was evaluated clinically and with the help of MRI. Following initial treatment, patients were included into our institution’s standardized gynecologic oncology follow-up program. For the first 3 years, patients were followed-up every 3 months, in the fourth and fifth year biannually, and yearly from the sixth to the tenth year after primary treatment. CT scans and MRI, and in selected cases [^18^F]FDG PET-CT scans, were performed on a yearly basis. Furthermore, if clinically suggested or in case of elevation of tumor markers, imaging was performed as indicated. Recurrent disease was either diagnosed clinically, by biopsy, or using imaging methods. Death and its cause were documented based on autopsy results and on the records in death certificates.

### Butyrylcholinesterase measurement

Blood samples (serum) for biochemical assessment were obtained as part of routine check-up prior to initiation of treatment. Serum BChE levels were determined by a kinetic enzyme assay by our institutional laboratory.

### Statistical analysis

Categorical variables are presented as numbers and proportions, continuous variables as medians (interquartile range, IQR). Group differences in categorical and continuous variables were analyzed using chi-square and Kruskal–Wallis tests, respectively. To assess associations between serum BChE levels and survival probabilities, we used the median serum BChE level to assign patients to risk groups of “low” and “high” serum BChE. In addition, the effects of log age (logarithmic transformation was used to make the data more conform to the normal distribution), BMI (<25 vs. >25 kg/m2), FIGO tumor stage (FIGO IV vs. III vs. II vs. I), radiological or pathological presence of lymph node metastases (periaortic [±pelvic] vs. pelvic vs. no lymph node metastases), histological grading (G3 vs. G2 vs. G1), histological subtype (squamous cell vs. non-squamous cell carcinoma), and the log tumor size on survival probabilities were assessed. Survival probabilities were calculated by the product limit method of Kaplan and Meier. Differences between groups were tested using the log-rank test. The results were analyzed for the endpoints progression-free (PFS), cancer-specific (CSS), and overall survival (OS). For PFS, events were defined as the date of progression, for CSS as cancer-related death, and for OS as cancer-related death and death due to any cause. Patients who were still alive were censored at the date of last follow-up. Multivariate Cox regression models for PFS, CSS, and OS were performed including all variables associated with survival in univariate analyses. Results of uni- and multivariate survival analyses are given as *p*-values (hazard-ratio [HR] and 95% confidence interval [95%CI]). *P*-values <0.05 were considered statistically significant. We used the statistical software IBM SPSS 24.0 for Mac (IBM Corp. Released 2016. IBM SPSS Statistics for MAC, Version 24.0., Armonk, NY, USA) for statistical analysis.

## Results

**Fig. 1:**
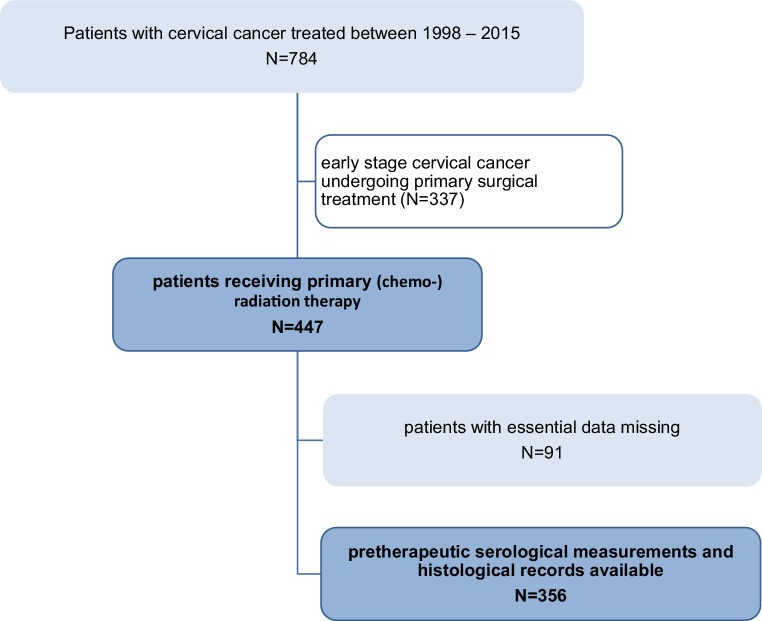
Flowchart of included and excluded patients

**Fig. 2:**
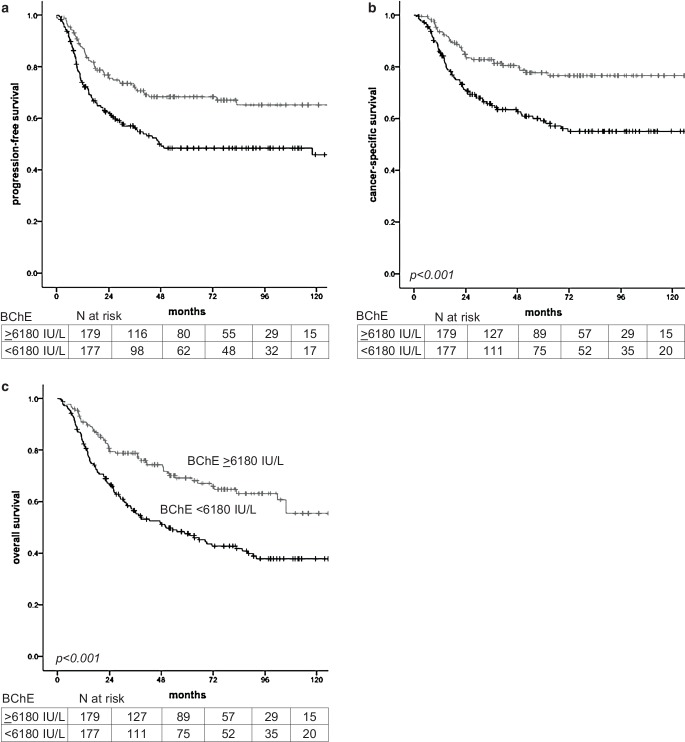
Kaplan–Meier curves for progression-free (**a**), cancer-specific (**b**), and overall survival (**c**) stratified for the risk groups serum butyrylcholinesterase (*BChE*) levels (< vs. ≥6180 IU/L)

In total, we identified 447 patients who were treated with primary (chemo-)radiation therapy for cervical cancer at our institution between 1998 and 2015 (Fig. 1). Of these, serological measurements of BChE prior to treatment were available in 356 patients, who were therefore eligible for the present study. Patients’ median (IQR) age at first diagnosis was 55.3 years (44.7–67.5). Based on pretreatment imaging, tumors had a median maximal diameter of 50 mm (36–60). In 220 patients (61.8%), regional lymph nodes were removed prior to (chemo-)radiation therapy, with pelvic and periaortic lymphadenectomy in 197 (55.3%) and 57 patients (16%), respectively. Apart from that, lymph nodes were evaluated with the help of pelvic MRI in combination with either abdominal CT or [^18^F]FDG PET-CT scans. In 260 patients (73%) a platinum-containing chemotherapy was given concurrently to primary radiation therapy, 96 (27%) were treated by radiation therapy without concurrent chemotherapy. All patients received EBRT, while the radiation field was extended to the periaortic region in 85 patients (24%). Following EBRT, 341 patients (95.7%) underwent IGABT. Within a median (IQR) follow-up time of 65.6 months (35.9–101.3), 105 (29.5%) and 54 patients (15.2%) experienced cancer-related and non-cancer related death, leading to 5‑year PFS, CSS, and OS of 58.3% (SE 2.8), 68.4% (SE 2.8), and 58.2% (2.8%), respectively. Median (IQR) time to recurrence was 12.1 months (7.6–23.2). Prior to treatment, patients had a median (IQR) serum BChE of 6180 IU/L (4990–7710). We evaluated differences in serum BChE levels between risk groups based on clinical and pathological parameters. Of note, lower serum BChE levels were associated with lower BMI (*p* < 0.001), advanced tumor stage (*p* = 0.04), poor response to treatment (*p* = 0.002), the occurrence of disease recurrence (*p* = 0.003), and the risk of death (*p* < 0.001). Patients’ characteristics and the respective median serum BChE levels are given in Table 1. To evaluate the association between low serum BChE and patient survival, we stratified patients into risk groups using the median serum BChE level of 6180 IU/L as a cut-off value. Results of uni- and multivariate survival analyses for PFS, CSS, and OS investigating the effects of serum BChE and other clinical and pathological parameters are given in Table 2. Of note, low serum BChE levels were associated with shorter PFS, CSS, and OS in both uni- and multivariate analyses, even after adjustment for the effects of other prognostic parameters. Other factors associated with shorter PFS, CSS, and OS were advanced FIGO tumor stage, larger tumor size, and the presence of lymph node metastases and of non-squamous cell carcinoma histology. However, apart from low serum BChE levels, the only factors independently associated with shorter PFS and CSS in multivariate analyses were an advanced FIGO tumor stage, a larger tumor size, and a non-squamous cell carcinoma histology. Factors independently associated with shorter OS were low serum BChE levels, advanced FIGO tumor stage, larger tumor size, presence of lymph node metastases, and a patient’s age. Kaplan–Meier curves for PFS, CSS, and OS stratified according to serum BChE levels are shown in Fig. 2.

**Table 1:** Patients’ characteristics and median pretreatment serum butyrylcholinesterase levels in 356 patients with cervical cancer treated with primary (chemo-)radiation therapy

	Number (%)	Median (IQR) serum BChE levels (IU/L)	*p*-value
*Age*			0.09^a^
<70 years	284 (79.8)	6280 (5010–7860)	
≥70 years	72 (20.2)	5930 (4790–7270)	
*BMI*			<0.001^b^
Underweight	18 (5.1)	5970 (4210–6700)	
Normal weight	126 (35.4)	5900 (4670–7030)	
Overweight/obese	148 (41.6)	6780 (5540–8030)	
NA	64 (18)	–	
*FIGO tumor stage*			0.04^b,c^
IB1	12 (3.4)	7500 (4790–7660)	
IB2	7 (2)		
IIA1	7 (2)	6490 (5080–7970)	
IIA2	4 (1.1)		
IIB	116 (32.6)		
IIIA	3 (0.8)	5960 (4810–7010)	
IIIB	142 (39.9)		
IVA	14 (3.9)	6520 (5080–8010)	
IVB	51 (14.3)		
*Histologic subtype*			0.1^b^
Squamous cell carcinoma	317 (89.0)	6220 (5020–7750)	
Adenocarcinoma	32 (9)	6200 (4870–7670)	
Others (clear cell, serous papillary)	7 (2)	4790 (4160–5920)	
*Histologic grading*			0.8^b^
G1	14 (3.9)	6010 (4510–7570)	
G2	190 (53.4)	6110 (4970–7580)	
G3	122 (34.3)	5060 (6180–7980)	
NA	30 (8.4)	–	
*Lymph node metastases* ^d^			0.3^b^
No	171 (48)	6340 (5040–7840)	
Pelvic only	124 (34.8)	6000 (4950–7200)	
Periaortic only	5 (1.4)	8650 (4150–9600)	
Pelvic and periaortic	45 (12.6)	6570 (5610–7630)	
NA	11 (3.2)	–	
*Tumor size*			0.1^a^
<40 mm	85 (23.9)	6340 (5070–7650)	
≥40 mm	227 (63.8)	6930 (4790–7220)	
NA	44 (12.3)	–	
*Response to treatment*			0.002^b^
Response	336 (94.4)	6250 (5030–7780)	
Persistent or progressive disease	20 (5.6)	5050 (3660–6420)	
*Recurrence*			0.003^a^
Yes	138 (38.8)	5620 (4810–7090)	
No	218 (61.2)	6400 (5070–7900)	
*Status at last observation*			<0.001^b^
Free of disease	173 (48.6)	6670 (5380–8030)	
Stable disease	5 (1.4)	7890 (5590–9670)	
Progressive disease	19 (5.3)	6500 (5120–8420)	
Non-cancer-related death	54 (15.2)	5920 (4470–7100)	
Cancer-related death	105 (29.5)	5560 (4690–6930)	

*BChE* butyrylcholinesterase*, IQR* interquartile range; *BMI* body mass index (underweight: BMI < 18.5 kg/m^2^; normal weight: BMI 18.5–24.9 kg/m^2^; overweight/obese: BMI ≥ 25 kg/m^2^), *NA* not available

^a^Mann–Whitney-U test

^b^Kruskal–Wallis test

^c^FIGO I vs II vs III vs IV

^d^Evaluated by surgical or radiological staging

**Table 2:** Association between clinico-pathological risk factors and low pretreatment serum butyrylcholinesterase and survival in 356 patients with cervical cancer treated with primary (chemo-)radiation therapy

	Progression-free survival				Cancer-specific survival				Overall survival			
	Univariate		Multivariate^b^		Univariate		Multivariate^b^		Univariate		Multivariate^b^	
	*P-*value^a^	HR (95% CI)^b^	*P-*value	HR (95% CI)	*P-*value^a^	HR (95% CI)^b^	*P-*value	HR (95% CI)	*P-*value^a^	HR (95% CI)^b^	*P-*value	HR (95% CI)
Log age	0.2^b^	0.6 (0.3–1.3)	–	–	0.3^b^	0.6 (0.3–1.5)	–	–	0.002^b^	2.8 (1.5–5.5)	0.004	2.7 (1.3–5.4)
BMI^c^	0.9	0.9 (0.7–1.3)	–	–	0.9	0.9 (0.6–1.4)	–	–	0.7	0.9 (0.6–1.2)	–	–
FIGO tumor stage^d^	<0.001	2.2 (1.7–2.7)	<0.001	2.6 (1.6–3.9)	<0.001	2.2 (1.7–2.8)	<0.001	2.8 (1.7–4.7)	<0.001	1.6 (1.3–2.0)	<0.001	2.3 (1.5–3.5)
Lymph node metastases^e^	0.001	1.8 (1.4–2.2)	0.1	0.7 (0.4–1.1)	<0.001	1.8 (1.4–2.3)	0.1	0.7 (0.4–1.1)	<0.001	1.8 (1.4–2.3)	0.04	0.7 (0.4–0.9)
Grading^f^	0.7	0.9 (0.8–1.2)	–	–	0.6	0.9 (0.7–1.2)	–	–	0.2	0.9 (0.7–1.1)	–	–
Histologic subtype^g^	0.004	1.9 (1.2–3)	0.04	1.6 (1.1–2.6)	0.01	1.9 (1.1–3.1)	0.07	1.6 (0.9–2.8)	0.2	1.3 (0.8–2.1)	–	–
Log Tumor size	<0.001^b^	2.7 (1.6–4.5)	0.04	1.7 (1.1–2.8)	<0.001^b^	3.0 (1.6–5.4)	0.04	1.8 (1.1–3.3)	0.001^b^	2.2 (1.4–3.6)	0.04	1.6 (1.1–2.6)
BChE^h^	<0.001	2.1 (1.4–3.2)	0.002	1.8 (1.2–2.6)	<0.001	2.8 (1.7–4.5)	0.001	2.2 (1.4–3.5)	<0.001	2.1 (1.5–3.1)	<0.001	2.04 (1.4–2.9)

*BChE *butyrylcholinesterase*, BMI* body mass index, *HR* hazard ratio, *CI* confidence interval

^a^Kaplan–Meier analysis

^b^Cox regression analyses

^c^Under- vs. normal- vs. overweight/obese

^d^FIGO IV vs. III vs. II vs. I

^e^Pathological or radiological (CT or PET-CT) determined periaortic (±pelvic) vs. pelvic vs. no lymph node metastases

^f^G3 vs. G2 vs. G1

^g^Squamous cell carcinoma (SCC) vs. Non-SCC

^h^BChE = butyrylcholinesterase < vs. ≥6180 IU/l

## Discussion

Accurate estimation of a patient’s individual oncological outcome is substantial for adequate counseling and planning of treatment. In the present study, we aimed to evaluate the role of pretreatment serum BChE levels in predicting survival in patients with cervical cancer treated with primary (chemo-)radiation therapy. Our results thereby revealed that low pretreatment serum BChE was a novel prognostic biomarker for PFS, CSS, and OS in patients with LACC and early cervical cancer with lymph node metastases, independent of possible confounding factors. Additionally, low serum BChE levels were associated with advanced tumor stage, poor response to treatment, and a risk of recurrence and death. To the best of our knowledge, this is the first study to evaluate the clinical significance of pretreatment serum BChE levels in these patients and to determine its value as an independent predictor for survival. As a glycoprotein enzyme produced by the liver, BChE hydrolyzes various exogenous choline esters. Mutations in the BChE gene leading to the protein’s deficiency result in abnormally slow metabolic degradation of exogenous choline ester drugs, such as the depolarizing neuromuscular blocking agent succinylcholine [18]. Furthermore, reductions in serum BChE can be found in various clinical conditions, such as injury, liver damage, infectious disease, and malnutrition [20]. During more recent years, a decrease in BChE could be observed in various malignancies [21–26, 33]. The most important finding of our study was the association between low serum BChE levels and shorter PFS, CSS, and OS. This association remained unchanged even after adjustment for the effects of other clinical parameters in multivariate analysis. Similarly to our results, in bladder, prostate, upper tract urothelial cancer, and terminally cancer patients with peritoneal carcinomatosis, low serum BChE levels were associated with shorter survival [22–24, 34]. In cervical cancer, estimation of oncological outcome is generally based on clinico-pathological parameters such as tumor stage, histological subtype, tumor size, lymphovascular space invasion, and lymph node involvement [7, 30, 32, 35]. Nevertheless, several biomarkers such as CRP and albumin were found to be associated with shorter survival in patients with cervical cancer, suggesting inflammatory reactions of the body to affect the individual’s oncological outcome [12, 36]. In addition, serum BChE levels were found to be inversely correlated with tumor stage, suggesting an increase in tumor load to either impair the enzyme’s production or to increase its consumption. Our results are in accordance with studies that reported similar findings in patients with oral and gastric cancer [21, 26]. Exact mechanisms underlying these findings are not yet elucidated. As the production of BChE and albumin in the liver occur in a coupled fashion, mechanisms leading to a decrease of serum BChE levels might be comparable to those leading to hypoalbuminemia [20]. While CRP is one of the major acute-phase proteins up-regulated within the hepatic response to the cytokine cascade, albumin decreases within acute injury or inflammation [37]. Likewise, an inverse association between acute-phase proteins, such as CRP and IL-6, and serum BChE levels has been reported [33, 38]. Therefore, lower BChE levels in advanced tumor stages may be caused by an underlying chronic infection. Furthermore, indirect evidence exists for a role of BChE in hydrolyzing acetylcholine (ACh) in addition to acetylcholinesterase [39]. Besides being one of the most important neurotransmitters, ACh and its receptors form the so-called non-neuronal cholinergic system (NNCS). A strong involvement of the NNCS in numerous cell functions including cell cycle, control of differentiation, apoptosis, angiogenesis, organization of the cytoskeleton, cell–cell contact, and migration is becoming more and more apparent [40]. Up-regulation of ACh was demonstrated to stimulate growth of tumor cell lines in lung cancer [41]. Similarly, nicotinic ACh receptors were found to be highly expressed in cervical cancer cell lines and their stimulation induced tumor cell proliferation in vitro [42]. Another interesting finding of our study was the correlation between pretreatment serum BChE levels and response to (chemo-)radiation therapy. Patients with response to treatment had significantly higher serum BChE levels than those who developed progressive disease during treatment. Our findings are supported by results of a study that investigated the change of serum BChE levels in patients with head and neck and cervical cancer during radiotherapy. A rise of serum BChE up to normal levels was thereby associated with a lasting response after 6 months follow-up [25]. In accordance with results of previous reports, in our cohort, serum BChE levels correlated with the patients’ BMI, with underweight patients having lower serum BChE levels than normal to overweight patients. BChE is regarded as sensitive marker for nutritional decline [33], a condition characteristic of advanced tumor load and shorter survival [11]. Strongly influenced by inflammation, a key contributing factor in the pathophysiology of protein–energy malnutrition, serum BChE levels sensitively decrease in the acute inflammatory phase and promptly increase when inflammation improves [34, 38]. Thereby, rather an inadequate availability of substrates for its synthesis as opposed to hepatocellular failure seems to be responsible for a decrease in BChE during malnutrition [34, 43].

Strengths of the present study include its relatively large sample size and standardized follow-up program, which allowed us to adequately evaluate response to treatment and the occurrence of recurrent/progressive disease. However, there are some limitations that deserve mention. Data were analyzed retrospectively, leading to shortcomings, including patient selection and incomplete data acquisition. In addition, due to the long study period, the type of treatment has changed over time, such as the addition of concurrent chemotherapy to radiation therapy in the majority of patients starting in 1999, the systematic use of IGABT since 2001 [31, 32], and the increasing use of surgical lymph node staging (pelvic and later periarotic).

## Conclusion

In conclusion, the results of the present study allow generation of the hypothesis that serum BChE is a possible novel, independent biomarker predicting PFS, CSS, and OS in patients with LACC and early stage cervical cancer with lymph node metastases treated with primary (chemo-)radiation therapy. In addition, we observed low serum BChE levels to be associated with advanced tumor load, poor response to treatment, and a low BMI. We thereby contribute to the understanding of BChE’s function and add a possibly valuable prognostic biomarker for patients with LACC that is cheap and easy to obtain, and therefore suitable for use in daily clinical practice. However, larger prospective studies are required to validate our results.
